# Effects of Strength Training on Neck Muscle Function and Tenderness in Patients with Chronic Headache: A Secondary Analysis of a Clinical Trial

**DOI:** 10.3390/jcm14207364

**Published:** 2025-10-17

**Authors:** Jordi Padrós-Augé, Gemma Victoria Espí-López, Henrik Winther Schytz, Karen Søgaard, Rafel Donat-Roca, Henrik Baare Olsen, Bjarne Kjeldgaard Madsen

**Affiliations:** 1 Department of Physiotherapy, University of Vic—Central University of Catalonia, Campus UManresa, 08242 Manresa, Spain; 2 Sport, Exercise and Human Movement, University of Vic—Central University of Catalonia, 08500 Vic, Spain; 3 Exercise Intervention for Health Research Group (EXINH-RG), Faculty of Physiotherapy, University of Valencia, Gasco Oliag St., 5, 46010 Valencia, Spain; 4 Department of Physiotherapy, Faculty of Physiotherapy, University of Valencia, Gasco Oliag St., 5, 46010 Valencia, Spain; 5 Danish Headache Centre, Department of Neurology, Faculty of Health Sciences, Glostrup Hospital, 2600 Copenhagen, Denmark; 6 Department of Sports Science and Clinical Biomechanics, University of Southern Denmark, 5230 Odense, Denmark; 7 Department of Clinical Research, University of Southern Denmark, 5000 Odense, Denmark

**Keywords:** chronic headache, migraine, tension-type headache, exercise therapy, strength training, muscle function

## Abstract

**Background/Objectives**: This study presents a secondary analysis from a previously published trial on strength training and postural correction in chronic headache patients. Here, we investigate changes in neck muscle function and tenderness, and their relationship with headache symptoms. **Methods**: A total of 22 headache patients from a single-arm open-label trial were included in this study to assess muscle function and tenderness. The maximum voluntary contraction of neck flexion and extension, shoulder elevation, and craniocervical flexion test were performed at baseline, week eight, and week 14. The extension/flexion ratio of the neck, the rate of force development, and the early rate of force development for shoulder elevation were calculated. Muscle tenderness was analyzed using the total tenderness score (TTS) and correlations between these outcomes and headache changes were explored. **Results**: After the intervention muscle tenderness significantly decreased (−5.6 ± 6.4; *p* < 0.001) and significant improvements in muscle function were observed. Correlations of muscle function showed a significant and moderate correlation between TTS and extension/flexion ratio (Spearman rho: 0.567, *p* = 0.014). **Conclusions**: The results indicate that strength training and postural correction improve muscle function and reduce pericranial tenderness in patients with chronic headaches. These findings suggest that muscle tenderness and extension/flexion ratio may be useful for monitoring exercise interventions focused on improving the strength and balance of the neck in patients with chronic headaches.

## 1. Introduction

Muscle tenderness (MT) in the pericranial region is one of the main peripheral mechanisms involved in the pathophysiology of primary headaches [1,2,3]. Increased MT has been associated with a higher frequency of headache episodes; however, the underlying mechanisms driving this increased sensitivity, as well as the most effective strategies to reduce it, are still not fully understood [4]. In tension-type headache (TTH), increased MT seems to play a role in activating the cervical trigeminal system, whereas in migraine, it can serve both as a trigger and a consequence of the migraine episode [5,6]. Given that, MT is more prominent in chronic compared to episodic headaches, and that similar levels of MT have been reported in patients with chronic neck pain and chronic headache. It is essential to further investigate the factors influencing MT and how changes in MT relate to the clinical expression of primary headaches.

Similarly, the function of the neck and shoulder muscles has been widely studied in patients with primary headaches. Previous studies have shown differences in the function of these muscles, suggesting increased weakness and reduced balance [7,8]. Furthermore, several authors have reported that impairments in cervical and shoulder function contribute to headache progression. For example, poor cervical function has been associated with neck pain, which at the same time is considered a poor prognostic factor in migraine [9,10].

The physiological basis for the relationship between the cervical region and headaches lies in the convergence of afferent inputs at the trigeminocervical complex [11]. According to this theory, nociceptive input originating from cervical structures can influence headache patterns. As a result, elevated MT in the cervical pericranial muscles may contribute to increased headache frequency and intensity [12]. Therefore, interventions aiming to reduce MT are of particular interest. Strength training (ST) is one of the most effective strategies to achieve this. In patients with chronic neck pain due to trapezius myalgia, high-intensity ST has been shown to reduce neck pain and improve muscle function [13].

The maximal force that a muscle group can produce during an isometric voluntary contraction is a critical functional indicator, and influences the relative workload experienced during daily tasks. Another important parameter is the ability to rapidly produce force, referred to as the rate of force development (RFD). The relation between this muscle functions and pain is described in several studies. Andersen et al. found that RFD in the trapezius muscle was reduced in patients with chronic neck pain, and that pain significantly impaired their ability to generate force quickly [14]. Similarly, Madsen et al. demonstrated that 10-week ST intervention improved RFD and reduced neck muscle pain in patients with chronic and frequent episodic TTH [15]. However, the benefits of strength training depend on adequate adherence and compliance. In the same study by Madsen et al., participants in the intervention group failed to achieve significant strength gains, suggesting insufficient training effort [16]. Andersen et al. further showed that participants with high or moderate adherence did not show meaningful improvements [17].

Other functional impairments have also been observed in patients with primary headache. Florencio et al. [18] reported statistically and clinically relevant differences in neck flexor and endurance tests between migraine patients and healthy controls. These findings align with other studies reporting reduced muscle thickness and poor function of the deep neck flexors in patients with primary headache [19].

An additional parameter that may reflect functional impairment is the ratio between cervical extension and flexion force, which has been found to be altered in patients with primary headache compared to healthy individuals [7,8]. Unbalanced muscles potentially change pattern activation and increase co-activation of neck muscles, which in turn may result in greater muscle overload and pain [20,21].

According to Henneman’s size principle, motor units with the lowest threshold are recruited first and derecruited last during muscle contraction [22,23]. Postural corrections are focused on reducing low-load overactivity in the neck muscles. In sustained low-load positions, smaller fibers are recruited continuously, which increases fatigue and pain, particularly in muscles such as the sternocleidomastoid and upper trapezius in individuals with forward head posture [18]. High-intensity ST of the shoulder aims to increase muscle capacity and reduce the relative load, thereby desensitizing muscle fibers and leading to reduced neck pain. Meanwhile, low load craniocervical training targets the improvement in the cervical balance and motor control of the neck [24]. We hypothesize that balancing cervical extension/flexion ratio and improving trapezius muscle function can reduce nociceptive input to the trigeminocervical complex and ultimately alleviate headache symptoms.

In a previous study [25], we observed that a 14-week strength training and postural correction program significantly reduced headache frequency and duration in some patients with chronic migraine and tension-type headache. This analysis aimed to analyze the effects of the intervention on muscle function and muscle tenderness and the relationship between muscle function and muscle tenderness in patients with chronic migraine and chronic tension-type headache. The hypothesis was that strength training and postural corrections are effective for improving muscle function, and muscle function improvements are correlated with decreased muscle tenderness and headache reduction variables.

## 2. Materials and Methods

This is the second manuscript presenting data related to muscle function and muscle tenderness from a previously conducted intervention study focused on posture correction and strength training. The first manuscript presented the headache-related outcomes from the same sample [25].

Measurements were collected at baseline, at the end of supervised period (week 8), and after a follow-up period (week 14) as part of a single-arm open-label trial conducted at the Danish Headache Center (DHC). A total of 22 patients with chronic primary headache participated. Assessments were carried out by a physiotherapist (JPA) at the DHC. The project was conducted in collaboration with the University of Southern Denmark under the guidance of Professor KS and engineer HB. Additionally, all procedures were supervised by an expert physiotherapist (BKM), who had previously used the same equipment.

### 2.1. Participants

Patients aged between 18 and 65 years with at least 5 headache days per 14 days at the moment of recruitment. For migraineurs, stable pharmacological treatment with either CGRP monoclonal antibodies or Onabotulinumtoxin A (defined as at least two consecutive doses of the same medication) was required. Exclusion criteria were pregnancy; post-traumatic headache, or headache that is likely to be associated with trauma; significant psychiatric comorbidities, such as severe depression; medication overuse headache diagnosis; severe arthrosis in the neck, shoulder, or disk herniation in the neck; other neurological diagnoses (i.e., multiple sclerosis). All patients were diagnosed by a neurologist following the 3rd edition of the International Classification of Headaches Disorders (ICHD-3) [26].

Patients were assigned to exercise groups based on their availability to attend weekly supervised sessions at the DHC. Outcome assessments were conducted at baseline, week 8, and week 14. Further details about the intervention are provided in a previous publication [25].

### 2.2. Outcomes

A set of tests were performed at three timepoints to assess changes in muscle function and pericranial muscle tenderness. Data collection was carried out before intervention (T0), at the end of the supervised training period (T1), and one month after the intervention ended (T2).

#### 2.2.1. Muscle Function

Maximal voluntary contractions (MVC) of cervical flexion, cervical extension, and shoulder elevation were measured using a computerized system consisting of a Vishay Nobel force transducer (type KIS-2, max. 2 kN; Vishay Precision Group, Karlskoga, Sweden) was connected to a signal conditioning unit (type PWR02, including a strain gauge amplifier SCC-SG24; National Instruments, Austin, TX, USA), and a data acquisition card (Daqcard-6036E; National Instruments, Austin, TX, USA). Signals were sampled at a frequency of 100 Hz and filtered using a low pass filter with a 10 Hz cut-off frequency. The TheR2Force software (Vishay Precision Group, Karlskoga, Sweden) was used for MVC data acquisition. All tests were conducted with the participant in a seated position without contacting the floor with their feet.

Moment arm measurements were calculated individually. For shoulder elevation, the distance between the C7 spinous process and the acromion was used. For neck extension, the moment arm was defined as the distance from C7 to the external occipital protuberance. For neck flexion, the dynamometer was positioned at the level of the eyebrows; adjustments to the dynamometer arm were recorded and added to or subtracted from the extension measurement to calculate the appropriate flexion moment arm.

**Figure 1:**
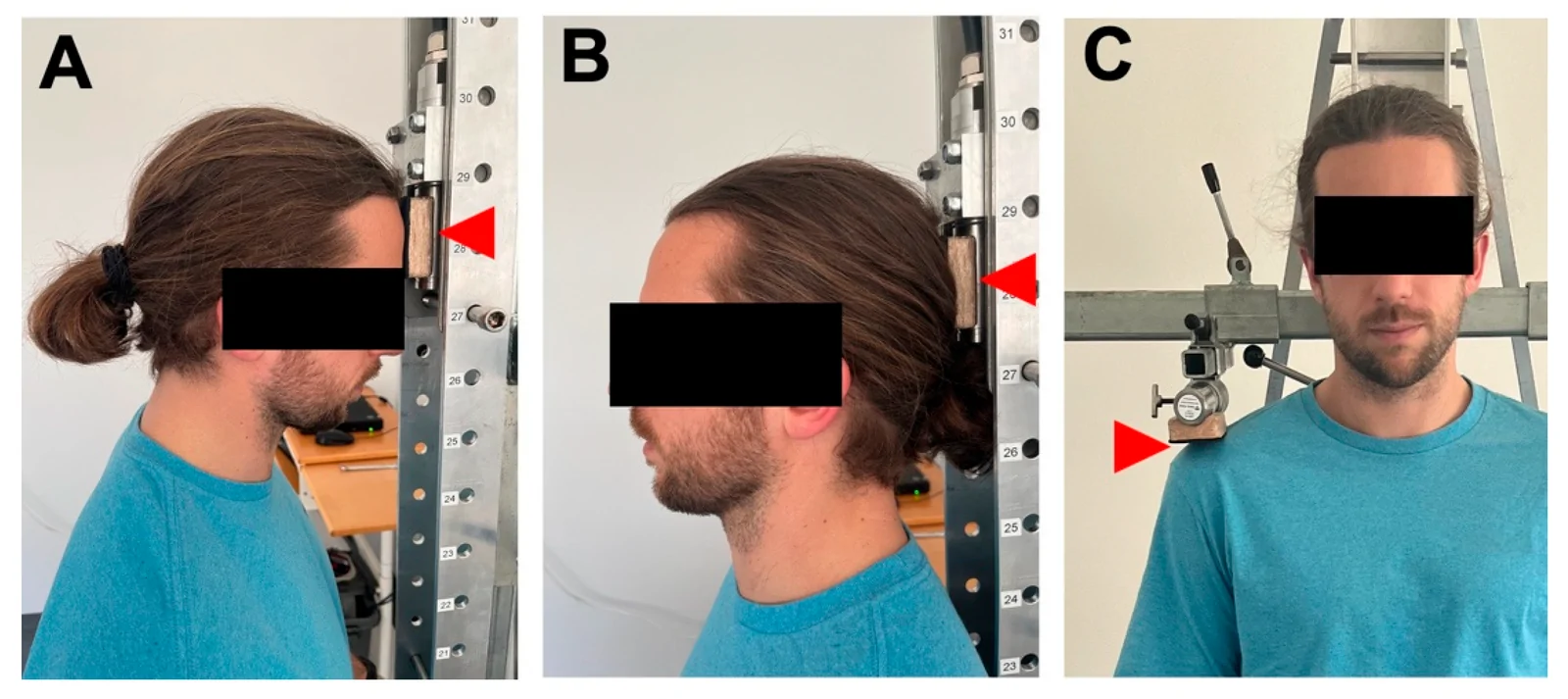
Testing procedure. (**A**) Neck flexion. (**B**) Neck Extension. (**C**) Shoulder elevation. The red arrow indicates the position of the force transducer.

Participants were instructed to perform three maximal isometric repetitions for each movement, with 60 s of rest between repetitions (Figure 1). This protocol has been used in previous research [27].

For neck measurements, participants were instructed to “push slowly and progressively until reaching maximum effort.” Verbal encouragement was provided during each trial. If the final repetition exceeded the peak force of the previous ones by more than 5%, up to two additional repetitions were performed. To ensure standardization, participants were instructed not to engage their abdominal muscles during the flexion tests (e.g., by raising the legs), nor their spinal extensors during extension (e.g., by arching the back or separating the back from the chair).

The moment for each movement was calculated as peak force*moment arm. The cervical extension/flexion ratio was computed by dividing the extension moment by the flexion moment.

Deep neck flexors (DNF) function was evaluated using the craniocervical flexion test (CCFT), which assesses the ability to recruit deep neck flexors while minimizing superficial muscle activation. The test uses a pressure biofeedback unit, and the CCFT score was determined using a 6-point scale based on the maximum pressure increment achieved without activation of superficial flexors: 0 = 20 mHg; 1 = 22 mHg; 2 = 24 mHg; 3 = 26 mHg; 4 = 28 mHg; 5 = 30 mHg) [28].

For shoulder elevation, participants were instructed to push against the force transducer “as hard and as fast as possible.” The rate of force development (RFD, in Nm/s) was calculated as the steepest slope over 100 ms in the rising phase of the filtered force–time curve. For each participant, the trial with the highest peak value was used for analysis. The early rate of force development (eRFD) was also calculated, representing the slope over the first 250 ms after contraction onset. Contraction onset was defined as the point at which force reached 2.5% of the peak value [29].

#### 2.2.2. Muscle Tenderness

Muscle tenderness was assessed using the Total Tenderness Score (TTS). This method involves palpation of eight bilateral anatomical sites: the masseter, frontalis, temporalis, trapezius, sternocleidomastoid, mastoid process, coronoid process, and the insertions of the occipital muscles. Each site was scored using a 4-point scale that combines behavioral and verbal responses, according to the following criteria: “0 = no pain or tenderness, “1 = mild pain or tenderness, “2 = moderate pain or tenderness” and “3 = severe pain or tenderness” [30]. Additionally, there are two subscales for the TTS: one referring to the neck region (TTS-neck), including four sites: trapezius, sternocleidomastoids, process mastoideus, and occipital muscles), and another related to the face area (TTS-face), including masseter, frontalis, temporalis and coronoid process.

### 2.3. Statistics

Headache and neck pain-related outcomes were analyzed and presented in a previous publication [25]. In the present analysis, muscle function data are presented as means, standard deviations (SD), and mean changes at endpoint (weeks 7–8) and follow-up (weeks 13–14), adjusted for baseline values. Subgroup analyses were conducted based on headache type. Due to the small sample size (*n* < 30) and non-normal data distribution, within-group comparisons were performed using the Wilcoxon signed-rank test, while between-group comparisons were conducted using the Mann–Whitney U test.

For analysis of the cervical extension/flexion ratio, a normal reference value of 1.7 was used, based on a previous study [31]. The absolute difference between the subject’s value and the normal value was used to measure the change in the extension/flexion ratio over time. A significance level of 0.05 has been defined for all statistical tests.

Missing data were handled by pairwise deletion. Only participants with valid paired measurements at both timepoints were included in each Wilcoxon signed-rank comparison.

Given the exploratory nature of the analysis and the small sample size, no imputation of missing values was performed.

Stata 17.0 version (Stata Corp LLC, College Station, TX, USA) was used to calculate pre-post results, JASP version 0.18.3 was used to calculate the correlations and generate plots and SankeyMATIC (https://sankeymatic.com/build/ accessed on 11 November 2024) was used to create the Sankey diagrams.

## 3. Results

**Figure 2:**
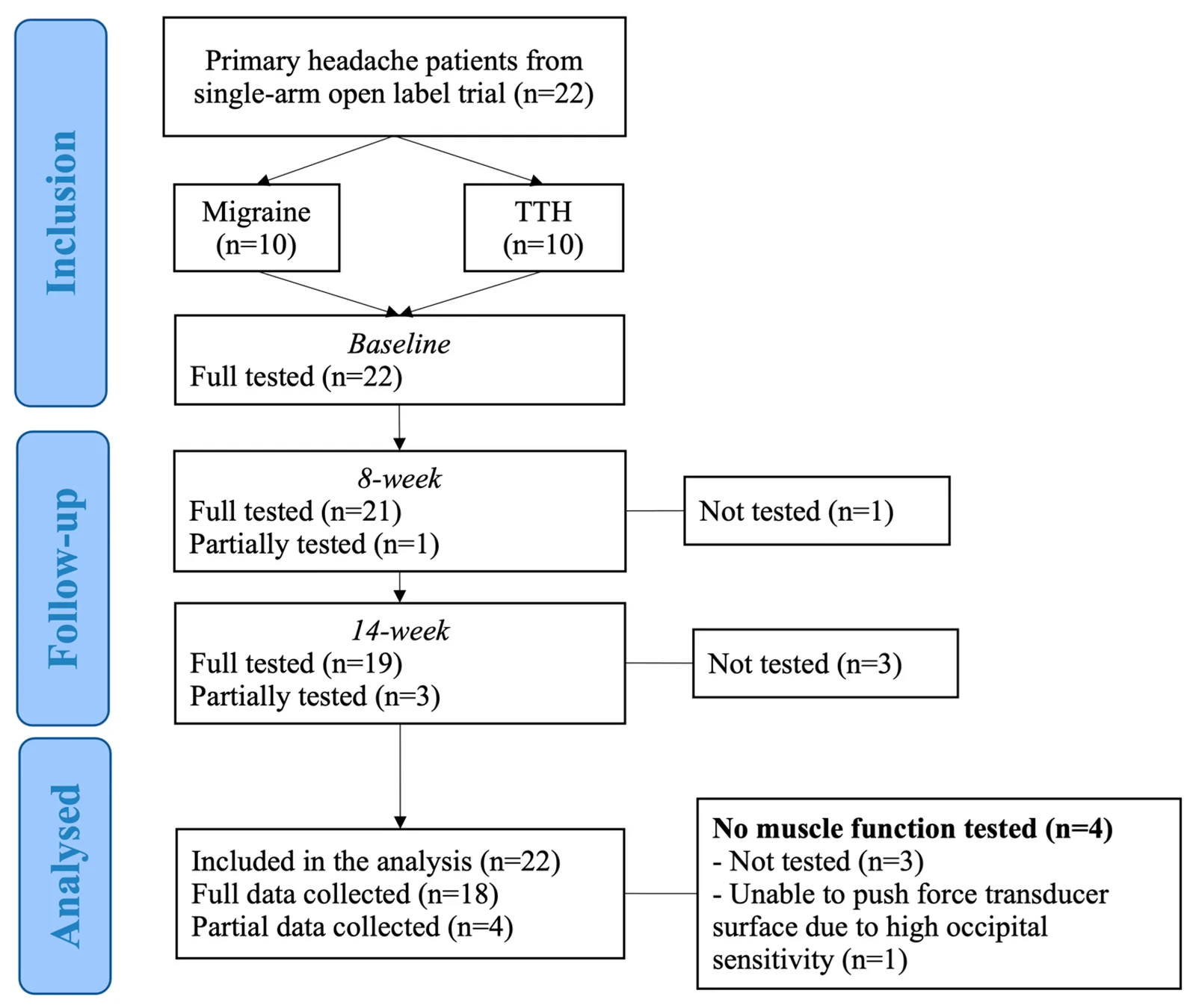
Flow chart of the study.

After completing an eight-week supervised strength training course and six additional weeks of home-training, a total of 22 patients with chronic migraine or chronic TTH were included in the analysis of muscle function and TTS (Figure 2).

One participant was unable to exert force against the transducer due to high occipital sensitivity and was excluded from the analysis. However, this participant’s ability to push increased from 39.14 N to 79.36 N at the endpoint, but was still limited due to occipital sensitivity. Another participant was not tested at the endpoint due to illness, and three additional participants were not tested at follow-up because holidays, sickness, and schedule incompatibilities.

### 3.1. Muscle Function

Muscle function was analyzed for the overall group and by subgroups regarding the headache form (Table 1). At baseline, only statistically significant differences between participants with migraine and those with TTH was observed in eRFD, which was lower in migraineurs (mean difference: 403.54 ± 161.23 N/s; *p =* 0.024). Overall muscle tenderness did not show significant improvement after 8 weeks; however, the TTS-neck subscale improved significantly (mean difference = −2.0 ± 3.6; *p* = 0.0102). At follow-up, muscle function variables continued to improve compared to baseline, except for RFD.

The TTS showed a significant reduction at follow-up (mean difference: −5.6 ± 6.4; *p* = 0.0001), and both subscales, TTS-neck and TTS-face, also decreased significantly.

Effect sizes (*r*) for within-group comparisons ranged from medium to large for most variables, indicating clinically relevant changes over time (Table 2). Statistically significant and large effects were observed for TTS and TTS-neck, CCFT, eRFD, and flexion and extension peaks after eight weeks. All muscle function variables resulted in a large effect after 14 weeks, except for elevation peak and RFD, while moderate effects were observed for elevation peak after 14 weeks.

**Figure 3:**
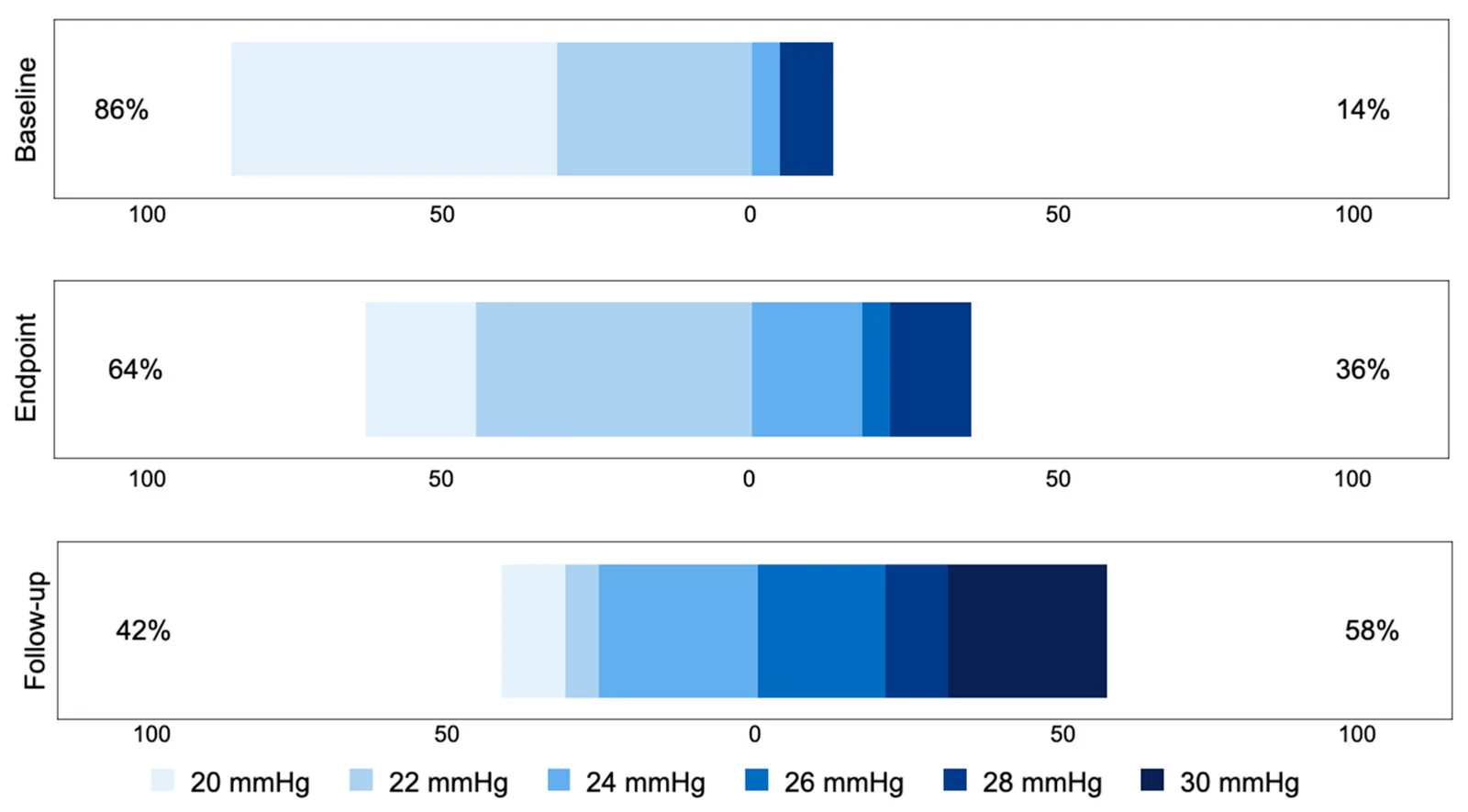
Likert plot for the craniocervical flexion test stages distribution at baseline, endpoint, and follow-up.

For CCFT score consistently improved during intervention. The distribution of participants across stages shifted markedly with an increase from 14% at baseline to 58% after 14 weeks in stages ≥ 26 mmHg (Figure 3).

### 3.2. Total Tenderness Progression

**Figure 4:**
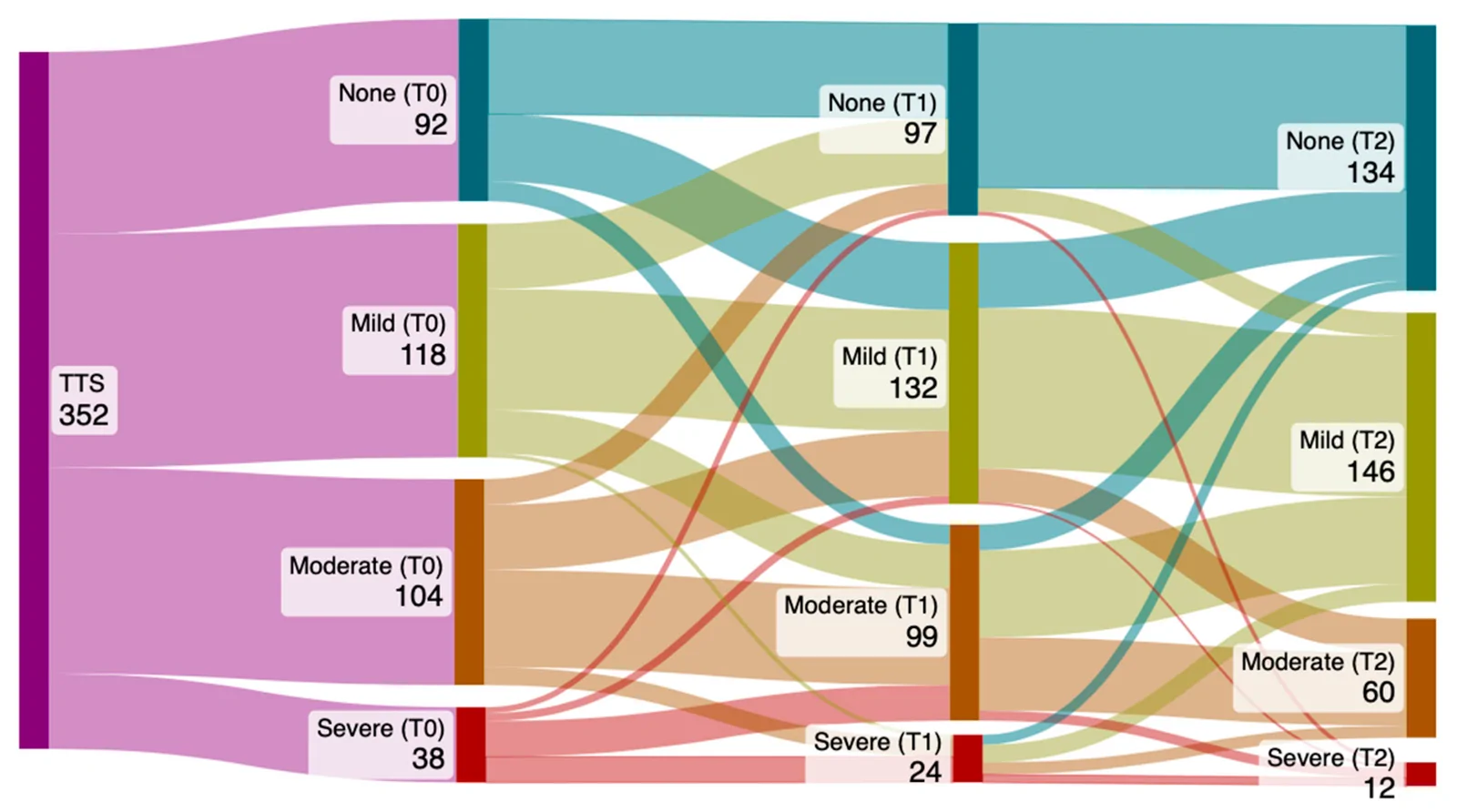
Sankey Diagram for the Total Tenderness Score progression. The extended version includes flows for each area (face/neck) and spots (see Supplementary Materials). TTS: Total Tenderness Score; T0: baseline; T1: weeks 7–8; T2: weeks 13–14.

The Sankey diagram (Figure 4) illustrates the progression of muscle tenderness based on the absolute values for TTS, its subscales (TTS-face and TTS-neck), and the individual scores of the eight spots at baseline, week 8, and week 14.

The proportion of non-tender spots increased from 26% at baseline to 38% at week 14. Sites rated as “mild” increased from 34% to 41%, while those rated “moderate” and “severe” decreased from 30% to 17% and from 11% to 3%, respectively. A detailed visualization of sensitivity progression for each region (neck/face) and for each of the eight spots is provided in the Supplementary Materials. Notably, three sites (SCM, trapezius superior, and coronoid process) did not show any cases rated as “severe” at follow-up.

### 3.3. Correlations Between Headache, Muscle Function and Tenderness

Correlation between changes in muscle function variables and changes in TTS was explored (Table 3). A statistically significant moderate correlation was found between the normalization of the extension/flexion ratio regarding TTS (Spearman rho: 0.567, *p* = 0.014). A weak association between the increase in eRFD and TTS; however, this was not statistically significant (Spearman rho: −0.218, *p* = 0.385). No significant correlations were found between RFD or CCFT and TTS. Additionally, a moderate correlation was observed between improvements in CCFT and normalization of the extension/flexion ratio (Spearman rho: 0.669, *p* = 0.002).

Correlations between headache frequency and duration reduction (previously reported [25]), muscle tenderness and muscle function were also analyzed (Table 4). A moderate correlation was found between the normalization of the neck extension/flexion ratio and the reduction in headache frequency (Spearman rho: −0.525, *p* = 0.025). A weak but non-significant correlation was observed between headache frequency and TTS (Spearman rho: −0.437, *p* = 0.062).

## 4. Discussion

This study analyzed, for the first time, the isolated effects of a combination of exercises on a set of muscle function outcomes and muscle tenderness. We hypothesized that the intervention would be effective in improving muscle function and reducing muscle tenderness. Furthermore, we proposed that improvements in muscle function would occur alongside or precede reductions in muscle tenderness, and that both changes would contribute to reductions in headache frequency and duration.

The results support the hypothesis that the intervention improve muscle function in both migraine and TTH patients. Significant improvements were observed through the intervention, including an 18% increase in eRFD, improvements for the CCFT Score, and normalization of the cervical extension/flexion ratio from 2.23 to 1.93 on average, which is discussed in depth below.

A clinically relevant and statistically significant reduction in TTS was found, particularly in the cervical region. This reduction was progressive across the three timepoints, reaching a 33% improvement by week 14. Notably, 70% of the overall reduction was attributed to decreased tenderness in the neck region (SCM, trapezius, suboccipital muscles, and mastoid process), while the remaining 30% occurred in the facial region (masseter, temporalis, frontalis, and coronoid process). This could be explained by improved muscle strength, leading to better muscle function and reduced relative workload, which in turn may decrease nociceptive input to the trigeminocervical complex, particularly from the upper trapezius and SCM. This may help explain the indirect reduction in tenderness across the entire pericranial area and its contribution to headache improvement [25].

The progression of tenderness over time, as illustrated in the Sankey diagram, showed a tendency toward decreasing muscle sensitivity after 8 weeks, with further improvement at 14 weeks. This suggests that continuing the intervention for a longer duration may yield additional reductions in tenderness.

The relationship between muscle function and tenderness was also explored, with notable findings. In particular, the normalization of the extension/flexion ratio was moderately correlated with reductions in tenderness. Improvements in CCFT were also correlated with normalization of the extension/flexion ratio. Although no direct correlation was found between CCFT score and TTS reduction. We interpret this as an indication that improved CCFT reflects better recruitment of deep and superficial neck flexors, reducing overactivity of the SCM. This aligns with the observed decrease in TTS for the SCM at follow-up. The extension/flexion ratio thus emerges as an important parameter for monitoring interventions targeting cervical muscle balance. As shown in previous studies, patients with headaches exhibit reduced steadiness during isometric cervical strength tests [16]; therefore, intervention should focus not only on strength gains but also on achieving muscular balance to reduce relative workload.

Regarding shoulder muscle function, the RFD did not improve significantly, whereas eRFD increased by 25% from baseline to follow-up. However, this was not significantly correlated with reductions in TTS. Previous studies on patients with chronic neck pain reported greater RFD improvements after 10 weeks of training, although they measured shoulder abduction [32]. In our sample, participants reported more days with neck pain and missed more exercise sessions due to headaches, which may partly explain the limited RFD improvement. Additionally, the follow-up period may have been too short to observe significant correlations between shoulder RFD and TTS reduction. Unlike other studies, our intervention included repeated TTS assessments, allowing us to observe the trajectory of tenderness reduction.

Finally, the analysis showed that normalization of the cervical extension/flexion ratio was associated with a reduction in headache frequency, suggesting that improving neck muscle balance could be a valuable therapeutic target for patients with chronic headache. In the study by Van Ettekoven et al., patients who performed neck retraction exercises during the follow-up period showed greater reductions in headache frequency, although the extension/flexion ratio was not assessed [24].

This study has some limitations. First is the open-label design with no control group, allowing comparability of muscle function and sensitivity to healthy controls and did not allow for assessor blinding. Second, a larger sample size would allow for better stratification by headache subtype or medication type.

Moreover, given the exploratory design and limited sample size, no correction for multiplicity was performed, and findings should therefore be interpreted with caution. and future analysis may consider robust statistical methods to test the relation between muscle function outcomes and pericranial tenderness and headache reduction.

In addition, certain methodological assumptions should be noted. A normative value of 1.7 was used for the cervical extension/flexion ratio. However, other studies have used different methods and reference values. For example, Benatto et al. used a handheld dynamometer secured with a non-elastic belt and conducted tests with participants in a supine position [7], whereas we conducted the tests using a stable setup and in a seated position, which we consider more functionally relevant. Therefore, instead of using the 1.9 reference value proposed by Benatto et al., we used a normative value from a study that also used seated testing conditions [31].

Unlike most studies, we calculated the extension/flexion ratio based on torque (moment of force) rather than raw peak force, offering greater accuracy and comparability across timepoints. Future research should replicate this methodology with larger sample sizes and consistent testing protocols to allow for multivariate regression analyses exploring the relationship between muscle function and headache outcomes.

## 5. Conclusions

This study showed that a strength training-based intervention was effective in improving muscle strength function and reducing the total tenderness score in a population with chronic headache. In addition, a relationship was found between changes in headache symptoms, muscle function, and muscle tenderness. Specifically, normalization of the cervical extension/flexion ratio was associated with a reduction in headache frequency. Therefore, improving cervical muscle balance should be considered a key goal in craniocervical training interventions for this population. Further studies with larger sample sizes and longer follow-up periods, using comparable muscle testing protocols, are needed to confirm these findings.

## Figures and Tables

**Table 1:** Mean values and mean differences for TTS and TTS-subscales for face and neck and muscle function outcomes at each timepoint. An extended version of Table 1 including each headache type is provided as a supplementary file.

	T0	T1	T2	Mean Changes			
	Mean ± SD	Mean ± SD	Mean ± SD	T1-T0	*p* (*)	T2-T0	*p* (*)
Overall group (n = 22); observations: 22 for TTS; 20 at endpoint and 17 at follow-up for RFD, eRFD, CCFT, and EFr.							
TTS	20.0 ± 10.2	17.9 ± 9.0	14.4 ± 9.0	−2.1; 5.9	0.055	−5.6; 6.4	<0.001 *
TTS face	9.1 ± 5.6	8.9 ± 4.8	7.6 ± 5.2	−0.2; 3.2	0.397	−1.5; 2.4	0.001 *
TTS neck	10.9 ± 5.5	8.9 ± 5.2	6.8 ± 5.4	−2.0; 3.6	0.010 *	−4.1; 5.3	<0.001 *
Extension peak	77.0 ± 31.2	91.2 ± 23.4	95.7 ± 27.5	13.4; 3.7	0.003 *	14.6; 3.9	0.002 *
Flexion peak	34.7 ± 9.8	49.9 ± 13.7	49.8 ± 11.4	16.4; 2.7	<0.001 *	15.3; 2.5	<0.001 *
Elevation peak	437.0 ± 144.6	477.9 ± 142.0	477.195 ± 171.7	54.6; 25.1	0.042 *	48.3; 24.0	0.062
EFr	2.23 ± 0.6	1.88 ± 0.4	1.93 ± 0.5	−0.32; 0.6	0.017 *	−0.5; 0.6	0.002 *
CCFT Score	0.77 ± 1.2	2.41 ± 1.4	2.95 ± 1.6	1.64; 1.7	<0.001 *	2.2; 1.7	<0.001 *
eRFD	958.7 ± 446.2	1129.9 ± 437.4	1165.6 ± 537.4	229.8; 418.5	0.012 *	242.2; 315.0	0.003 *
RFD	760.7 ± 653.2	827.2 ± 456.2	814.4 ± 611.4	148.4; 502.7	0.101	119.3; 774.4	0.267

TTS: total tenderness score; EFr: ratio between cervical extension and flexion; CCFT: craniocervical flexion test; eRFD; early Rate of Force Development; RFD: rate of force development. T1-T0: weeks 7–8 vs. baseline; T2-T0: weeks 13–14 vs. baseline. * Within-group significant differences (<0.05). Mean changes are expressed as the mean difference and standard error.

**Table 2:** Size effects of the intervention TTS, TTS-subscales face and neck and muscle function outcomes.

	T1-T0				T2-T0			
	Z	*p* (*)	*r*	Magnitude	Z	*p* (*)	*r*	Magnitude
	Overall group (n = 22); Observations: 22 for TTS; 20 at endpoint and 17 at follow-up for RFD, eRFD, CCFT and EFr.							
TTS	−2.419	0.143	0.516	Large	−3.685	0.001 *	0.786	Large
TTS face	−0.779	0.9252	0.166	Small	−2.987	0.014 *	0.637	Large
TTS neck	−3.198	0.0213 *	0.682	Large	−3.441	0.002 *	0.734	Large
Extension peak	−3.397	<0.001 *	0.760	Large	−2.485	0.011 *	0.603	Large
Flexion peak	−3.910	<0.001 *	0.853	Large	−3.479	<0.001 *	0.844	Large
Elevation peak	−1.929	0.055	0.421	Medium	−2.059	0.039 *	0.499	Medium
EFr	−1.941	0.053	0.434	Medium	−2.580	0.008 *	0.626	Large
CCFT Score	−3.880	<0.001 *	0.827	Large	−3.743	<0.001 *	0.859	Large
eRFD	−2.651	0.006 *	0.593	Large	−2.438	0.013 *	0.591	Large
RFD	−1.307	0.202	0.292	Small	−0.308	0.782	0.075	Small

TTS: total tenderness score; EFr: ratio between cervical extension and flexion; CCFT: craniocervical flexion test; eRFD: early Rate of Force Development; RFD: rate of force development. T1-T0: weeks 7–8 vs. baseline; T2-T0: weeks 13–14 vs. baseline. * Within-group significance differences (<0.05).

**Table 3:** Correlation between sensitivity and muscle function.

Variable		EFr	eRFD	RFD	CCFT
TTS	Spearman’s rho	0.567 *	−0.218	−0.092	0.152
	*p*-value	0.014	0.385	0.716	0.534

TTS: Total tenderness score; eRFD: early Rate of Force Development; RFD: Rate of Force Development; EFr: Ratio between neck extension and neck flexion; CCFT: Craniocervical flexion test score. * *p* < 0.05.

**Table 4:** Correlation between headache and TTS and muscle function.

Variable		TTS	EFr	eRFD	RFD	CCFT
HA Freq	Spearman’s rho	−0.437	−0.525 *	0.135	0.112	−0.369
	*p*-value	0.062	0.025	0.593	0.660	0.120
HA Dur	Spearman’s rho	−0.153	−0.133	0.320	0.309	0.101
	*p*-value	0.533	0.597	0.196	0.212	0.680

TTS: Total tenderness score; eRFD: early Rate of Force Development; RFD: Rate of Force Development; EFr: Ratio between neck extension and neck flexion; CCFT: Craniocervical flexion test score. * *p* < 0.05.

## Data Availability

The raw data supporting the conclusions of this article will be made available by the authors on request.

## Supplementary Materials

The following supporting information can be downloaded at: https://www.mdpi.com/article/10.3390/jcm14207364/s1, Table S1a: Effects of the intervention on muscle function on migraine patients; Table S1b: Effects of the intervention on muscle function on tension-type headache patients; Figure S1: Sankey diagram for TTS-face sites progression; Figure S2: Sankey diagram for TTS-neck sites progression; Figure S3: Sankey diagram for TTS-Masseter site progression; Figure S4: Sankey diagram for TTS-Coronoid site progression; Figure S5: Sankey diagram for TTS-Temporalis site progression; Figure S6: Sankey diagram for TTS-Frontalis site progression; Figure S7: Sankey diagram for TTS-Mastoid site progression; Figure S8: Sankey diagram for TTS-SCM site progression; Figure S9: Sankey diagram for TTS-Suboccipital site progression; Figure S10: Sankey diagram for TTS-Trapezius superior site progression.

## Abbreviations

The following abbreviations are used in this manuscript:

MT	Muscle Tenderness
TTH	Tension-Type Headache
TTS	Total Tenderness Score
ST	Strength Training
RFD	Rate of Force Development
eRFD	Early Rate of Force Development
CCFT	Craniocervical Flexion Test
MVC	Maximal Voluntary Contractions
DNF	Deep Neck Flexors
ICHD-3	International Classification of Headache Disorders 3rd edition.
