# Correction: Padrós-Augé et al. Effects of Strength Training on Neck Muscle Function and Tenderness in Patients with Chronic Headache: A Secondary Analysis of a Clinical Trial. *J. Clin. Med.* 2025, *14*, 7364

**DOI:** 10.3390/jcm15166465

**Published:** 2026-08-21

**Authors:** Jordi Padrós-Augé, Gemma Victoria Espí-López, Henrik Winther Schytz, Karen Søgaard, Rafel Donat-Roca, Henrik Baare Olsen, Bjarne Kjeldgaard Madsen

**Affiliations:** 1 Department of Physiotherapy, University of Vic—Central University of Catalonia, Campus UManresa, 08242 Manresa, Spain; rdonat@umanresa.cat; 2 Sport, Exercise and Human Movement, University of Vic—Central University of Catalonia, 08500 Vic, Spain; 3 Exercise Intervention for Health Research Group (EXINH-RG), Faculty of Physiotherapy, University of Valencia, Gasco Oliag St., 5, 46010 Valencia, Spain; 4 Department of Physiotherapy, Faculty of Physiotherapy, University of Valencia, Gasco Oliag St., 5, 46010 Valencia, Spain; 5 Danish Headache Centre, Department of Neurology, Faculty of Health Sciences, Glostrup Hospital, 2600 Copenhagen, Denmark; henrik.winther.schytz.01@regionh.dk (H.W.S.); bjarne.kjeldgaard.madsen@regionh.dk (B.K.M.); 6 Department of Sports Science and Clinical Biomechanics, University of Southern Denmark, 5230 Odense, Denmark; ksogaard@health.sdu.dk; 7 Department of Clinical Research, University of Southern Denmark, 5000 Odense, Denmark; hbolsen@health.sdu.dk

## Table Legend

In the original publication [1], there was a mistake in the legend for Table 2: “Mean changes are expressed in mean difference and standard error.” Since the values of the table are referred to Wilcoxon signed rank test and not mean difference and standard error. The correct legend appears below:

“TTS: total tenderness score; EFr: ratio between cervical extension and flexion; CCFT: craniocervical flexion test; eRFD: early Rate of Force Development; RFD: rate of force development. T1-T0: weeks 7–8 vs. baseline; T2-T0: weeks 13–14 vs. baseline. * Within-group significance differences (<0.05)”.

## Text Correction

There was a textual error in the original publication [1], the last paragraph of Section 3.3: “The Sankey diagram (Figure 4) illustrates the progression of muscle tenderness, the total values of the TTS, the TTS subscales (face and neck), and for each of the eight spots assessed in the TTS at three timepoints”.

This text should be removed since it is a duplicate explanation of the first paragraph of Section 3.2.

## Error in Table

In the original publication [1], there was a mistake in Table 1, Table S1a and Table S1b as published. The values indicate standard errors not standard deviations, so “±” is not correct for this column and should be corrected with “;”. The corrected Table 1, Table S1a and Table S1b appear below.

**Table 1:** Mean values and mean differences for TTS and TTS-subscales for face and neck and muscle function outcomes at each timepoint. An extended version of Table 1 including each headache type is provided as a supplementary file.

	T0	T1	T2	Mean Changes			
	Mean ± SD	Mean ± SD	Mean ± SD	T1-T0	*p* (*)	T2-T0	*p* (*)
Overall group (n = 22); observations: 22 for TTS; 20 at endpoint and 17 at follow-up for RFD, eRFD, CCFT, and EFr.							
TTS	20.0 ± 10.2	17.9 ± 9.0	14.4 ± 9.0	−2.1; 5.9	0.055	−5.6; 6.4	<0.001 *
TTS face	9.1 ± 5.6	8.9 ± 4.8	7.6 ± 5.2	−0.2; 3.2	0.397	−1.5; 2.4	0.001 *
TTS neck	10.9 ± 5.5	8.9 ± 5.2	6.8 ± 5.4	−2.0; 3.6	0.010 *	−4.1; 5.3	<0.001 *
Extension peak	77.0 ± 31.2	91.2 ± 23.4	95.7 ± 27.5	13.4; 3.7	0.003 *	14.6; 3.9	0.002 *
Flexion peak	34.7 ± 9.8	49.9 ± 13.7	49.8 ± 11.4	16.4; 2.7	<0.001 *	15.3; 2.5	<0.001 *
Elevation peak	437.0 ± 144.6	477.9 ± 142.0	477.195 ± 171.7	54.6; 25.1	0.042 *	48.3; 24.0	0.062
EFr	2.23 ± 0.6	1.88 ± 0.4	1.93 ± 0.5	−0.32; 0.6	0.017 *	−0.5; 0.6	0.002 *
CCFT Score	0.77 ± 1.2	2.41 ± 1.4	2.95 ± 1.6	1.64; 1.7	<0.001 *	2.2; 1.7	<0.001 *
eRFD	958.7 ± 446.2	1129.9 ± 437.4	1165.6 ± 537.4	229.8; 418.5	0.012 *	242.2; 315.0	0.003 *
RFD	760.7 ± 653.2	827.2 ± 456.2	814.4 ± 611.4	148.4; 502.7	0.101	119.3; 774.4	0.267

TTS: total tenderness score; EFr: ratio between cervical extension and flexion; CCFT: craniocervical flexion test; eRFD; early Rate of Force Development; RFD: rate of force development. T1-T0: weeks 7–8 vs. baseline; T2-T0: weeks 13–14 vs. baseline. * Within-group significant differences (<0.05). Mean changes are expressed as the mean difference and standard error.

**Table S1a:** Effects of the intervention on muscle function on migraine patients.

	T0	T1	T2	Mean changes			
	Mean ± SD	Mean ± SD	Mean ± SD	T1-T0	P (*)	T2-T0	P (*)
**Migraine (n = 10); *Observations:*** *10 for TTS; 10 at endpoint and 8 at follow up for the RFD, eRFD, CCFT and EFr.*							
**TTS**	22.6 ± 11.1	21.0 ± 8.9	17.0 ± 9.1	-1.6; 5.6	0.194	**-5.6 ; 5.6^a^**	**0.004**
**TTS face**	11.2 ± 6.4	10.8 ± 5.1	10.3 ± 5.4^b^	- 0.4; 3.1	0.345	- 0.9 ; 3.2	0.130
**TTS neck**	11.4 ± 5.2	10.2 ± 4.2	6.7 ± 5.3	- 1.2; 3.2	0.130	**- 4.7 ; 5.4^a^**	**0.014**
**Extension peak**	64.1 ± 18.0	87.2 ± 17.1	88.7 ± 24.2	0.24; 10.62	0.922	**24.4 ; 8.74^a^**	**0.047**
**Flexion peak**	32.0 ± 8.5	47.3 ± 9.1	49.1 ± 6.7	**15.43; 5.1^a^**	**0.014**	**16.47 ; 2.17^a^**	**0.016**
**Elevation peak**	348.8 ± 90.3^b^	421.6 ± 120.5	387.2 ± 110.8	72.8; 34.3	0.063	38.4; 36.1	0.323
**EFr**	2.2 ± 0.5	1.9 ± 0.24	1.8 ± 0.4	- 0.3; 0.6	0.090	**- 0.5 ; 0.6^a^**	**0.037**
**CCFT Score**	0.75 ± 1.2	2.3 ± 1.6	3.0 ± 2.1	1.6; 0.9	0.057	**2.3 ; 0.8^a^**	**0.031**
**eRFD**	728.1 ± 207.3^b^	997.6 ± 362.4	880.2 ± 313.1^b^	**269.6; 433.8^a^**	**0.050**	**197.7 ; 262.5^a^**	**0.047**
**RFD**	565.2 ± 361.7	725.5 ± 448.7	689.3 ± 571.1	160.2; 506.0	0.185	145.0 ; 663.4	0.292

**TTS**: Total tenderness score; **EFr**: Ratio between cervical extension and flexion; **CCFT**: Craniocervical flexion test; **eRFD**; early Rate of Force Development; **RFD**: Rate of force development. **T1-T0**: weeks 7-8 vs baseline; **T2-T0**: weeks 13-14 vs baseline; **MD**: Mean difference

*^a^* With-group differences significance (< 0.05). Mean changes are expressed in mean difference and standard error.

**Table S1b:** Effects of the intervention on muscle function on tension-type headache patients.

	T0	T1	T2	Mean changes			
	Mean ± SD	Mean ± SD	Mean ± SD	T1-T0	P (*)	T2-T0	P (*)
**Tension-type headache (n = 12); *Observations:*** *12 for TTS; 12 at endpoint and 11 at follow-up for RFD, eRFD, CCFT and EFr.*							
**TTS**	17.8 ± 9.3	15.3 ± 8.6	12.1 ± 8.8	- 2.5; 6.3	0.099	**- 5.7 ; 7.2^a^**	**0.010**
**TTS face**	7.4 ± 4.3	7.3 ± 4.4	5.2 ± 3.6^b^	- 0.1; 3.5	0.500	**- 2.2 ; 3.9^a^**	**0.025**
**TTS neck**	10.4 ± 6.0	7.5 ± 6.0	6.9 ± 5.7	**- 2.5; 3.9^a^**	**0.025**	**- 3.5 ; 5.5^a^**	**0.016**
**Extension peak**	86.7 ± 36.0	94.4 ± 27.9	100.6 ± 29.9	**23.13; 6.39^a^**	**0.027**	6.47 ; 15.1	0.769
**Flexion peak**	36.8 ± 10.5	52.0 ± 16.8	50.2 ± 14.1	**15.30; 3.31^a^**	**0.004**	**11.05 ; 5.5^a^**	**0.049**
**Elevation peak**	510.6 ± 142.3^b^	529.1 ± 145.7	540.2 ± 183.1	18.5; 41.6	0.665	29.6; 50.2	0.569
**EFr**	2.4 ± 0.7	1.9 ± 0.5	2.0 ± 0.5	- 0.4; 0.7	0.062	**- 0.5 ; 0.7^a^**	**0.021**
**CCFT Score**	0.8 ± 1.2	2.6 ± 1.2	2.9 ± 1.2	**1.8; 0.5^a^**	**0.005**	**2.1 ; 0.5^a^**	**0.002**
**eRFD**	1131.6 ± 504.6^b^	1238.2 ± 479.4	1365.5 ± 583.4^b^	197.4; 423.8	0.077	**273.4 ; 357.6^a^**	**0.019**
**RFD**	907.3 ± 791.2	910.5 ± 466.3	902.0 ± 653.0	138.6; 524.4	0.201	101.3 ; 878.6	0.362

**TTS**: Total tenderness score; **EFr**: Ratio between cervical extension and flexion; **CCFT**: Craniocervical flexion test; **eRFD**; early Rate of Force Development; **RFD**: Rate of force development. **T1-T0**: weeks 7-8 vs baseline; **T2-T0**: weeks 13-14 vs baseline; **MD**: Mean difference

*^a^* With-group differences significance (< 0.05). Mean changes are expressed in mean difference and standard error.

## Wrong Citation

In the Section 2.1 1st paragraph:

The current form is: “All patients were diagnosed by a neurologist following the 3rd edition of the International Classification of Headaches Disorders (ICHD-3) [25]”.

And should be:

“All patients were diagnosed by a neurologist following the 3rd edition of the International Classification of Headaches Disorders (ICHD-3) [26]”.

In the Section 2.2.1 2nd paragraph:

The current form is: “Moment arm measurements were calculated individually. For shoulder elevation, the distance between the C7 spinous process and the acromion was used. For neck extension, the moment arm was defined as the distance from C7 to the external occipital protuberance. For neck flexion, the dynamometer was positioned at the level of the eyebrows; adjustments to the dynamometer arm were recorded and added to or subtracted from the extension measurement to calculate the appropriate flexion moment arm [26]”.

Should be without reference at the end of the paragraph, since reference 27 appear later and all the procedure is related to this reference:

“Moment arm measurements were calculated individually. For shoulder elevation, the distance between the C7 spinous process and the acromion was used. For neck extension, the moment arm was defined as the distance from C7 to the external occipital protuberance. For neck flexion, the dynamometer was positioned at the level of the eyebrows; adjustments to the dynamometer arm were recorded and added to or subtracted from the extension measurement to calculate the appropriate flexion moment arm”.

The authors state that the scientific conclusions are unaffected. These corrections were approved by the Academic Editor. The original publication has also been updated.
