# Supplementary material for: Effects of Strength Training on Neck Muscle Function and Tenderness in Patients with Chronic Headache: A Secondary Analysis of a Clinical Trial
Source: J Clin Med. 2025 Oct 17;14(20):7364. doi: 10.3390/jcm14207364 (PMC12565405; doi:10.3390/jcm14207364)
Supplement: Supplementary file 1 [file jcm-14-07364-s001.zip › jcm-3905358-supplementary.pdf]

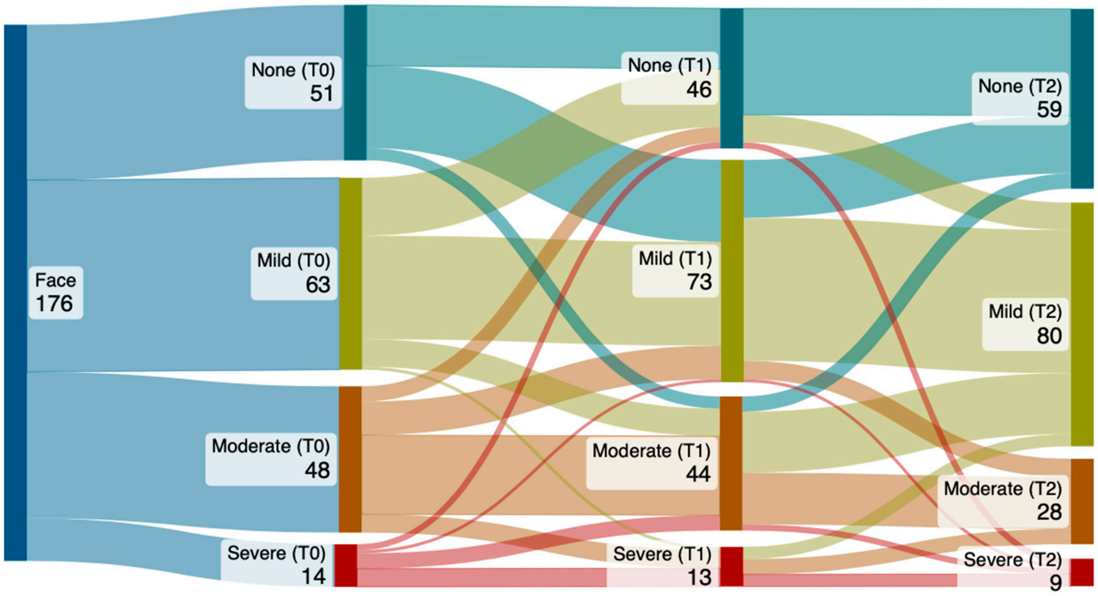


**Figure S1**. Sankey diagram for TTS-face sites progression. **TTS**: Total tenderness score; **T0**: baseline; **T1**: weeks 7-8; **T2**: weeks 13-14

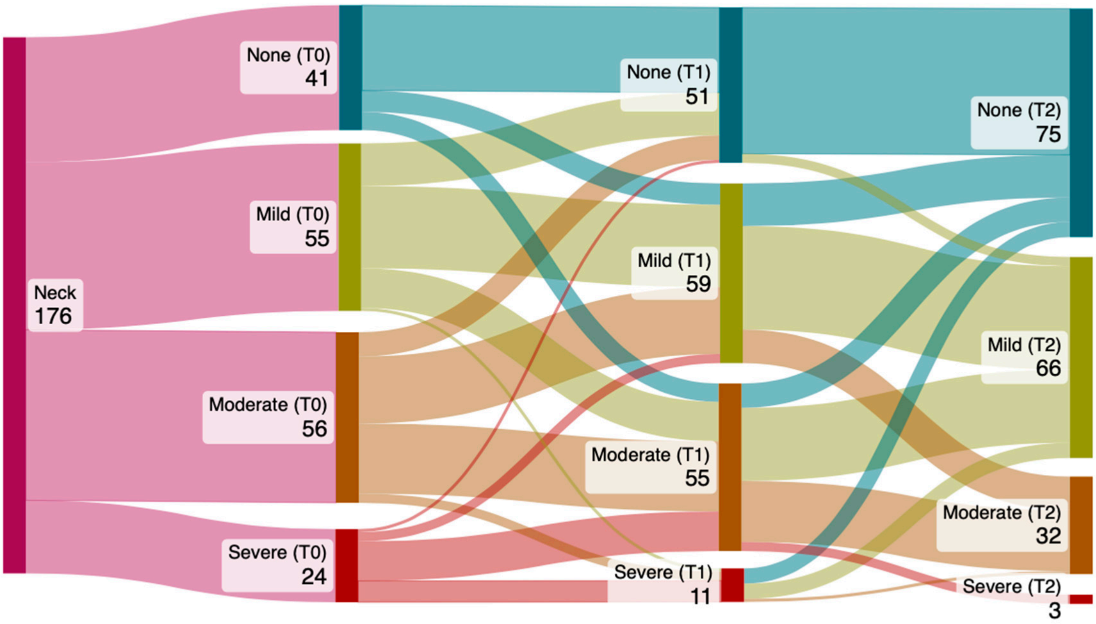


**Figure S2**. Sankey diagram for TTS-neck sites progression. **TTS**: Total tenderness score; **T0**: baseline; **T1**: weeks 7-8; **T2**: weeks 13-14

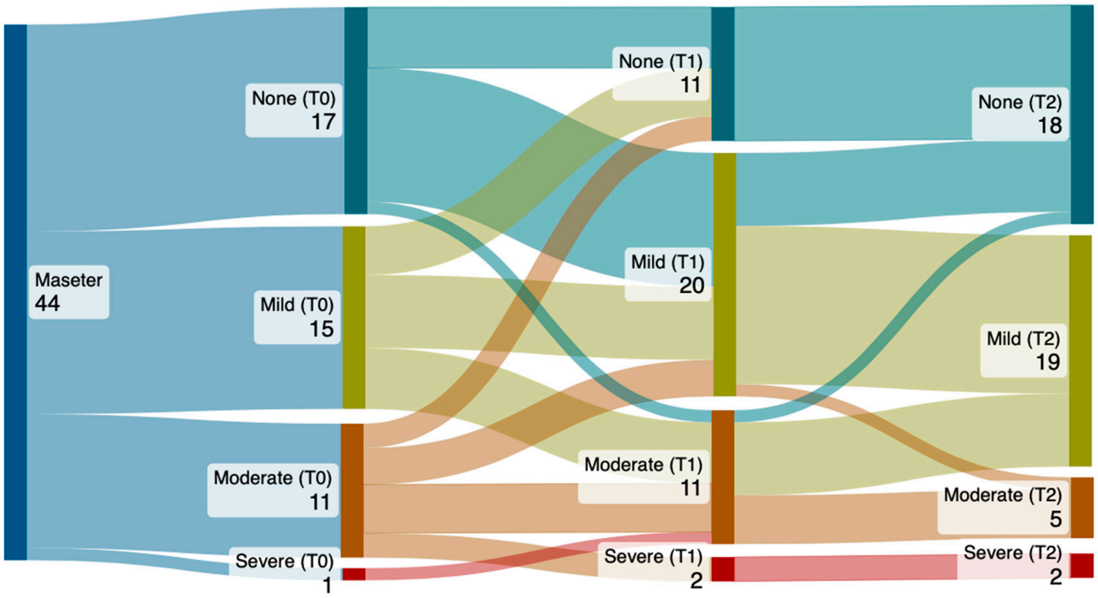


**Figure S3**. Sankey diagram for TTS-Masseter site progression. **TTS**: Total tenderness score; **T0**: baseline; **T1**: weeks 7-8; **T2**: weeks 13-14

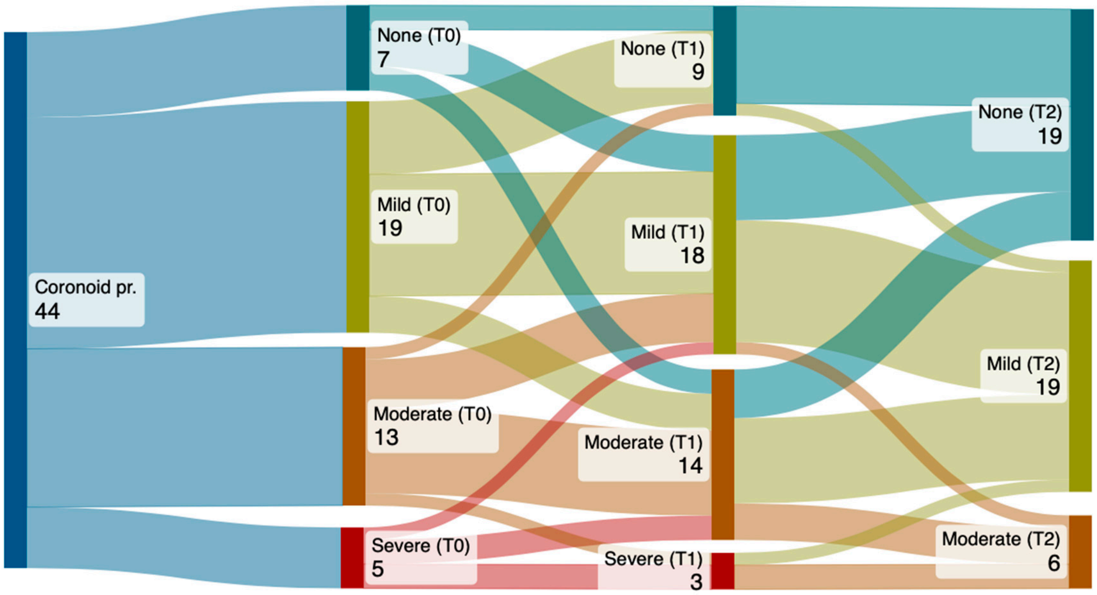


**Figure S4**. Sankey diagram for TTS-Coronoid site progression. **TTS**: Total tenderness score; **T0**: baseline; **T1**: weeks 7-8; **T2**: weeks 13-14

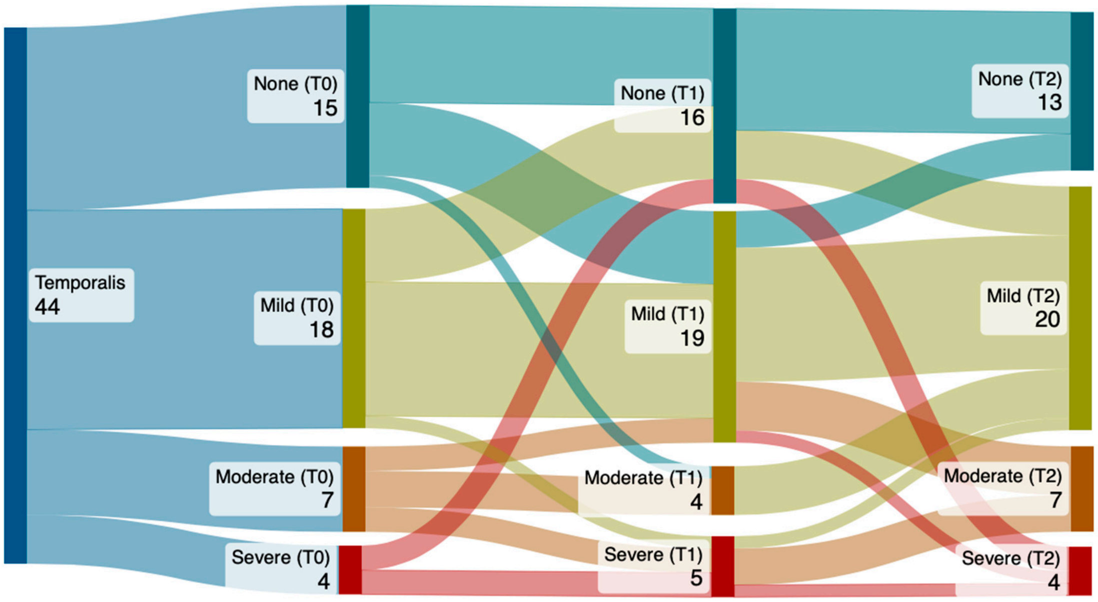


**Figure S5**. Sankey diagram for TTS-Temporalis site progression. **TTS**: Total tenderness score; **T0**: baseline; **T1**: weeks 7-8; **T2**: weeks 13-14

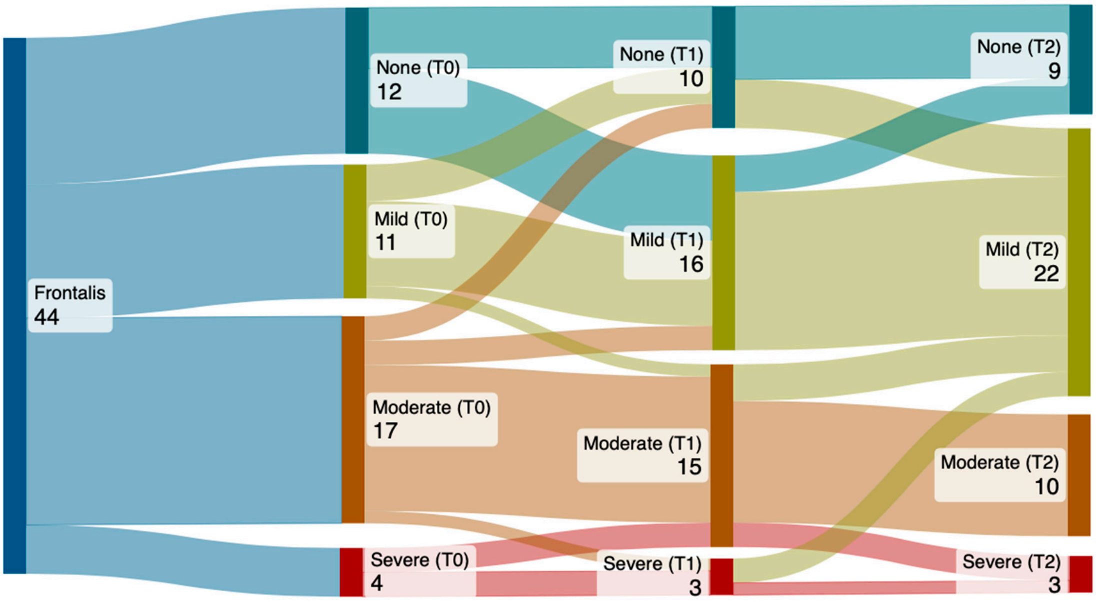


**Figure S6**. Sankey diagram for TTS-Frontalis site progression. **TTS**: Total tenderness score; **T0**: baseline; **T1**: weeks 7-8; **T2**: weeks 13-14

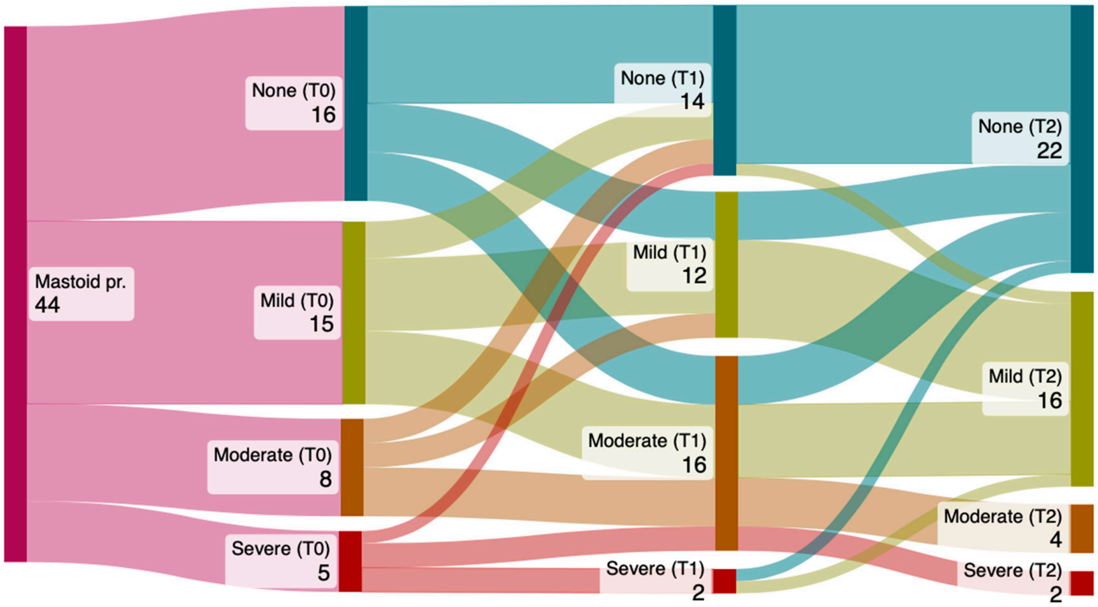


**Figure S7**. Sankey diagram for TTS-Mastoid site progression. **TTS**: Total tenderness score; **T0**: baseline; **T1**: weeks 7-8; **T2**: weeks 13-14

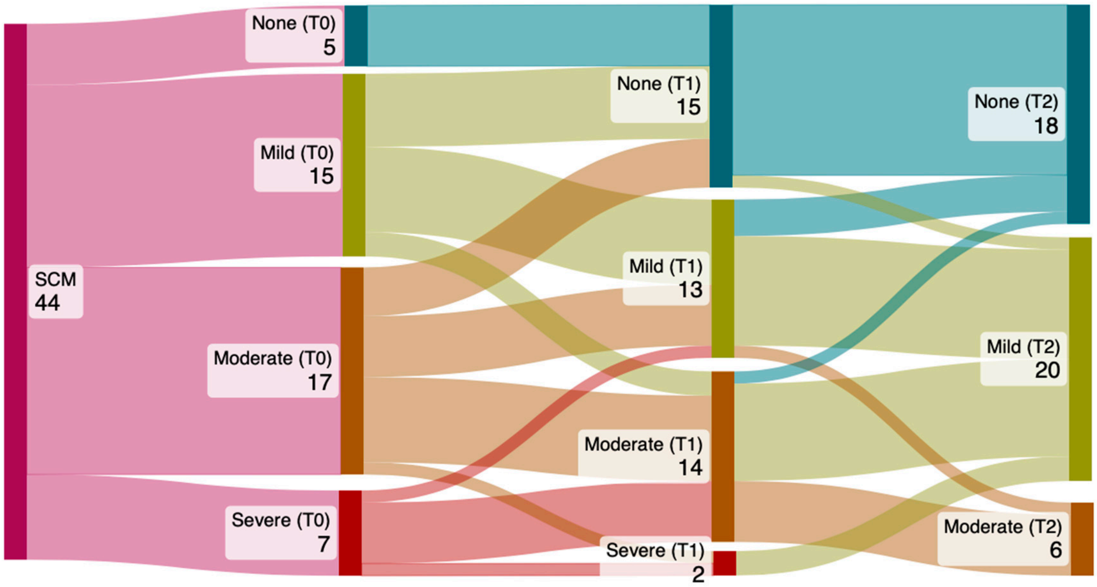


**Figure S8**. Sankey diagram for TTS-SCM site progression. **TTS**: Total tenderness score; **T0**: baseline; **T1**: weeks 7-8; **T2**: weeks 13-14

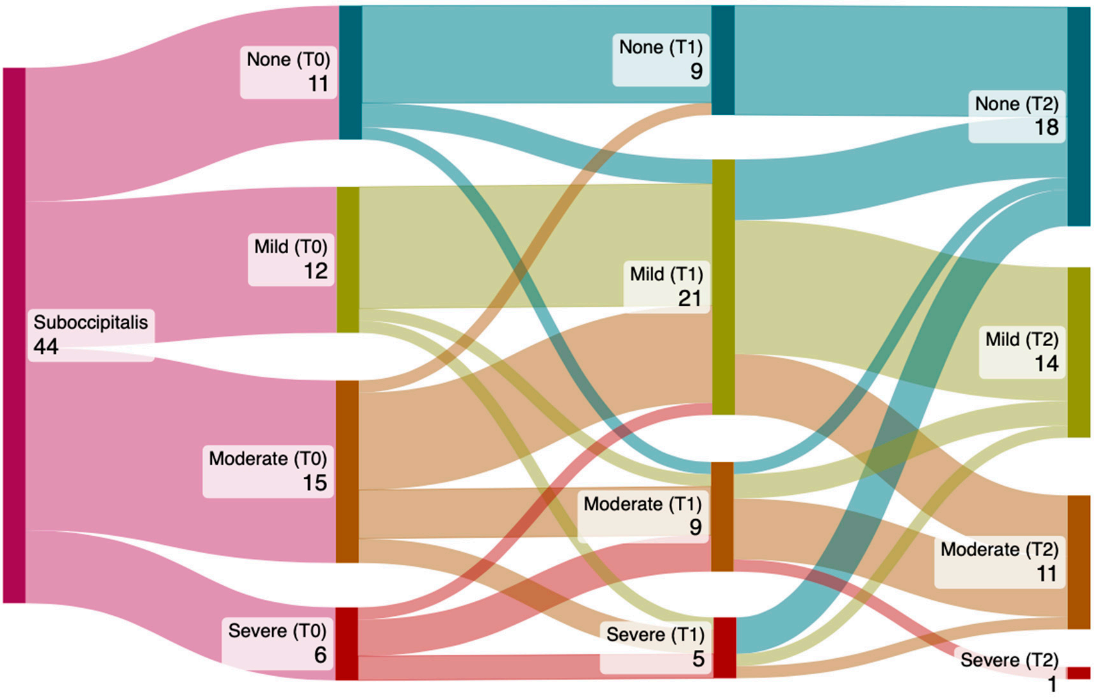


**Figure S9**. Sankey diagram for TTS-Suboccipital site progression. **TTS**: Total tenderness score; **T0**: baseline; **T1**: weeks 7-8; **T2**: weeks 13-14

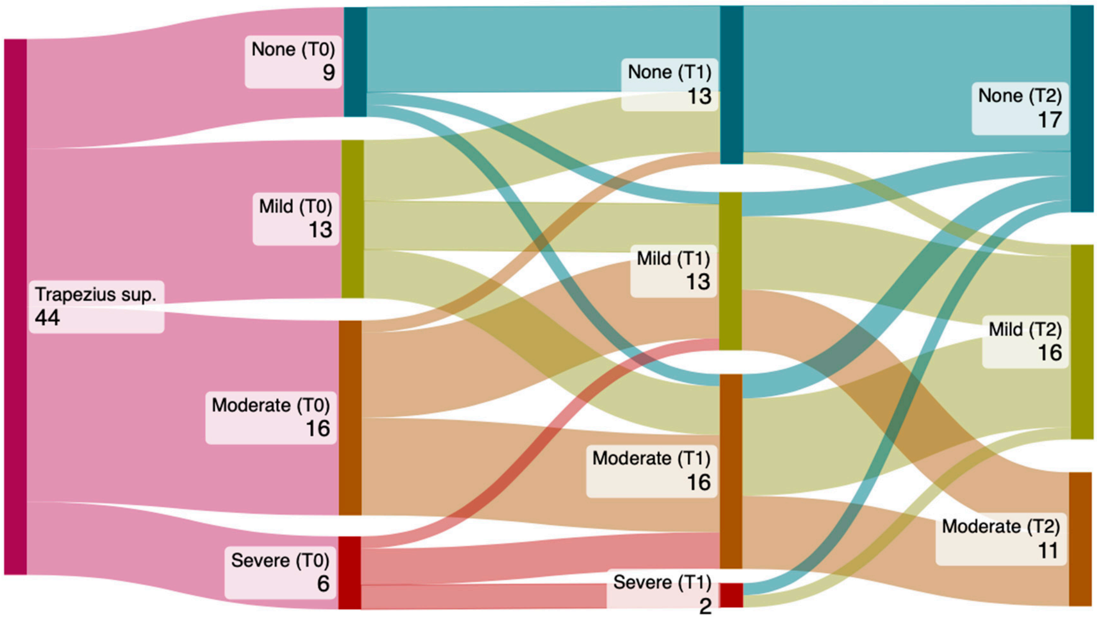


**Figure S10**. Sankey diagram for TTS-Trapezius superior site progression. **TTS**: Total tenderness score; **T0**: baseline; **T1**: weeks 7-8; **T2**: weeks 13-14

**Table S1a**. Effects of the intervention on muscle function on migraine patients.

| | T0 | T1 | T2 | Mean changes | | | |
|---|---|---|---|---|---|---|---|
| | Mean ± SD | Mean ± SD | Mean ± SD | T1-T0 | *P (*)* | T2-T0 | *P (*)* |
| **Migraine (n = 10);** ***Observations:*** *10 for TTS; 10 at endpoint and 8 at follow up for the RFD, eRFD, CCFT and EFr.* | | | | | | | |
| **TTS** | 22.6 ± 11.1 | 21.0 ± 8.9 | 17.0 ± 9.1 | - 1.6; 5.6 | 0.194 | **-5.6 ± 5.6**[a] | **0.004** |
| **TTS face** | 11.2 ± 6.4 | 10.8 ± 5.1 | 10.3 ± 5.4[b] | - 0.4; 3.1 | 0.345 | - 0.9 ± 3.2 | 0.130 |
| **TTS neck** | 11.4 ± 5.2 | 10.2 ± 4.2 | 6.7 ± 5.3 | - 1.2; 3.2 | 0.130 | **- 4.7 ± 5.4**[a] | **0.014** |
| **Extension peak** | 64.1 ± 18.0 | 87.2 ± 17.1 | 88.7 ± 24.2 | 0.24; 10.62 | 0.922 | **24.4 ± 8.74**[a] | **0.047** |
| **Flexion peak** | 32.0 ± 8.5 | 47.3 ± 9.1 | 49.1 ± 6.7 | **15.43; 5.1**[a] | **0.014** | **16.47 ± 2.17**[a] | **0.016** |
| **Elevation peak** | 348.8 ± 90.3[b] | 421.6 ± 120.5 | 387.2 ± 110.8 | 72.8; 34.3 | 0.063 | 38.4; 36.1 | 0.323 |
| **EFr** | 2.2 ± 0.5 | 1.9 ± 0.24 | 1.8 ± 0.4 | - 0.3; 0.6 | 0.090 | **- 0.5 ± 0.6**[a] | **0.037** |
| **CCFT Score** | 0.75 ± 1.2 | 2.3 ± 1.6 | 3.0 ± 2.1 | 1.6; 0.9 | 0.057 | **2.3 ± 0.8**[a] | **0.031** |
| **eRFD** | 728.1 ± 207.3[b] | 997.6 ± 362.4 | 880.2 ± 313.1[b] | **269.6; 433.8**[a] | **0.050** | **197.7 ± 262.5**[a] | **0.047** |
| **RFD** | 565.2 ± 361.7 | 725.5 ± 448.7 | 689.3 ± 571.1 | 160.2; 506.0 | 0.185 | 145.0 ± 663.4 | 0.292 |

**TTS**: Total tenderness score; **EFr**: Ratio between cervical extension and flexion; **CCFT**: Craniocervical flexion test; **eRFD**; early Rate of Force Development; **RFD**: Rate of force development. **T1-T0**: weeks 7-8 vs baseline; **T2-T0**: weeks 13-14 vs baseline; **MD**: Mean difference

[a] With-group differences significance (< 0.05). Mean changes are expressed in mean difference and standard error.

**Table S1b**. Effects of the intervention on muscle function on tension-type headache patients.

| | T0 | T1 | T2 | Mean changes | | | |
|---|---|---|---|---|---|---|---|
| | Mean ± SD | Mean ± SD | Mean ± SD | T1-T0 | *P (*)* | T2-T0 | *P (*)* |
| **Tension-type headache (n = 12);** ***Observations:*** *12 for TTS; 12 at endpoint and 11 at follow-up for RFD, eRFD, CCFT and EFr.* | | | | | | | |
| **TTS** | 17.8 ± 9.3 | 15.3 ± 8.6 | 12.1 ± 8.8 | - 2.5; 6.3 | 0.099 | **- 5.7 ± 7.2**[a] | **0.010** |
| **TTS face** | 7.4 ± 4.3 | 7.3 ± 4.4 | 5.2 ± 3.6[b] | - 0.1; 3.5 | 0.500 | **- 2.2 ± 3.9**[a] | **0.025** |
| **TTS neck** | 10.4 ± 6.0 | 7.5 ± 6.0 | 6.9 ± 5.7 | **- 2.5; 3.9**[a] | **0.025** | **- 3.5 ± 5.5**[a] | **0.016** |
| **Extension peak** | 86.7 ± 36.0 | 94.4 ± 27.9 | 100.6 ± 29.9 | **23.13; 6.39**[a] | **0.027** | 6.47 ± 15.1 | 0.769 |
| **Flexion peak** | 36.8 ± 10.5 | 52.0 ± 16.8 | 50.2 ± 14.1 | **15.30; 3.31**[a] | **0.004** | **11.05 ± 5.5**[a] | **0.049** |
| **Elevation peak** | 510.6 ± 142.3[b] | 529.1 ± 145.7 | 540.2 ± 183.1 | 18.5; 41.6 | 0.665 | 29.6; 50.2 | 0.569 |
| **EFr** | 2.4 ± 0.7 | 1.9 ± 0.5 | 2.0 ± 0.5 | - 0.4; 0.7 | 0.062 | **- 0.5 ± 0.7**[a] | **0.021** |
| **CCFT Score** | 0.8 ± 1.2 | 2.6 ± 1.2 | 2.9 ± 1.2 | **1.8; 0.5**[a] | **0.005** | **2.1 ± 0.5**[a] | **0.002** |
| **eRFD** | 1131.6 ± 504.6[b] | 1238.2 ± 479.4 | 1365.5 ± 583.4[b] | 197.4; 423.8 | 0.077 | **273.4 ± 357.6**[a] | **0.019** |
| **RFD** | 907.3 ± 791.2 | 910.5 ± 466.3 | 902.0 ± 653.0 | 138.6; 524.4 | 0.201 | 101.3 ± 878.6 | 0.362 |

**TTS**: Total tenderness score; **EFr**: Ratio between cervical extension and flexion; **CCFT**: Craniocervical flexion test; **eRFD**; early Rate of Force Development; **RFD**: Rate of force development. **T1-T0**: weeks 7-8 vs baseline; **T2-T0**: weeks 13-14 vs baseline; **MD**: Mean difference

[a] With-group differences significance (< 0.05). Mean changes are expressed in mean difference and standard error.
